# Identification of transcription factors regulating starch biosynthesis in maize through integrated GWAS and transcriptomic analysis

**DOI:** 10.1186/s12870-026-08557-z

**Published:** 2026-03-13

**Authors:** Jienan Han, Ran Li, Qianqian Liu, Ze Zhang, Guo Li, Zhennan Xu, Zhiqiang Zhou, Jianfeng Weng, Zhuanfang Hao, Degui Zhang, Hongjun Yong, Xinhai Li, Mingshun Li

**Affiliations:** 1 State Key Laboratory of Crop Gene Resources and Breeding, Institute of Crop Sciences, Chinese Academy of Agricultural Sciences, Beijing, 100081 China; 2 College of Agriculture, Heilongjiang Bayi Agricultural University, Daqing, 163319 China

**Keywords:** Maize, Kernel starch content, Transcription factor, Correlation analysis, Favourable haplotype, Starch biosynthesis gene

## Abstract

**Background:**

Kernel starch content (SC) is a major contributor to maize grain yield and end-use quality, but its genetic improvement is limited by incomplete understanding of genes linking natural variation to starch accumulation during endosperm development. Here, we aimed to identify starch-associated transcription factors (TFs) with breeding potential by integrating multi-environment phenotyping, genome-wide association studies (GWAS), transcriptome profiling of SC-contrasting subpopulations, haplotype analysis, and functional characterisation of key candidate regulators.

**Results:**

Kernel SC showed broad variation across four environments, ranging from 56.33% to 75.35%, with significant correlations among environments (*r* = 0.27–0.56, *P* < 0.01). Lines from the BSSS subpopulation exhibited consistently higher SC than those from the PA subpopulation, with increasing of 0.82%–2.79%. Comparative transcriptomic analysis of developing endosperm identified significant expression differences in starch biosynthesis genes, including 18 genes downregulated (0.41–0.90-fold) and four genes upregulated (1.23–1.74-fold) in BSSS. Several transcription factors (TFs) identified by GWAS showed coordinated expression with starch biosynthesis genes; seven of these were differentially expressed between BSSS and PA subpopulations and significantly correlated with multiple starch biosynthesis genes. Haplotype analysis showed that favourable Hap1 haplotypes of *ZmMYB71*, *ZmMYB4*, and *ZmGNAT16* were enriched in BSSS and were associated with a 1.51%–3.30% increase in kernel SC. Functional assays revealed that ZmMYB71 acts as a negative regulator: its overexpression suppressed key starch biosynthesis genes, *Sh1*, *Sh2*, and *GBSSI*, reduced AGPase, GBSS, and SSS activities by 3.26%–21.39%, and decreased kernel SC by 1.70%–4.91%. Conversely, *ZmMYB71* loss-of-function mutants showed upregulation of starch biosynthesis genes, increased enzyme activities (5.79%–17.20%), and increased kernel SC (2.67%–5.92%).

**Conclusions:**

This study identifies major transcriptional regulators underlying natural variation in maize kernel SC by integrating multi-environment phenotyping, GWAS, transcriptome profiling, and haplotype analysis. ZmMYB71, ZmMYB4, and ZmGNAT16 were prioritized as key candidate TFs associated with SC variation, and functional validation confirmed ZmMYB71 as a regulator of starch accumulation. Favourable haplotypes of these TFs were associated with increased kernel SC, providing practical targets for marker-assisted selection and genetic improvement of starch content in maize.

**Supplementary Information:**

The online version contains supplementary material available at 10.1186/s12870-026-08557-z.

## Background

Maize (*Zea mays* L.) is a staple crop for global food, feed, and biofuel production. The economic and nutritional value of maize kernels is largely determined by kernel starch content (SC), which typically accounts for 65%–75% of kernel dry weight and is a major contributor to grain yield [1]. Therefore, elucidating the genetic and molecular mechanisms controlling starch accumulation in developing kernels remains a central goal in maize biology and breeding.

Starch biosynthesis in the endosperm is a complex, multi-step process that begins with sucrose transport into the endosperm via specific sugar transporters such as ZmSWEET14c [2]. Sucrose synthase (SuSy) then cleaves sucrose to generate substrates for ADP-glucose pyrophosphorylase (AGPase) [3], which catalyses ADP-glucose formation [4, 5]. ADP-glucose subsequently serves as the substrate for starch synthases (SSSs), starch branching enzymes (SBEs), and debranching enzymes (DBEs), which collectively generate amylose and amylopectin [6–10]. Additional enzymes, including disproportionating enzyme (DPE) and starch phosphorylase (PHO), further contribute to starch metabolism and remodelling [11].

Beyond this core pathway, recent studies have uncovered post-translational regulatory mechanisms that fine-tune enzymatic activity. For example, a phosphomimetic mutation at Ser31 of the Sh2 subunit markedly enhances AGPase activity, and phosphoproteomic profiling has identified phosphorylation as a major regulatory mechanism modulating multiple starch biosynthetic enzymes, including SSIIIa, BEI, and BEIIb, particularly under thermal stress [12, 13]. Beyond post-translational control, functional insights have expanded beyond catalytic roles. In wheat and barley, SSIII not only elongates glucan chains but also serves as a scaffolding protein that organises multi-enzyme complexes, while dynamic GBSSI localisation enables real-time adjustment of amylose synthesis [14, 15]. Consistent with these broader functions, mutations in *Bt2*, which encodes an AGPase small subunit, disrupt basal endosperm transfer layer development in maize, revealing an unexpected role for AGPase in coordinating carbohydrate flux and seed filling [16].

In parallel with biochemical regulation, starch accumulation in the endosperm is governed by a transcriptional network that controls the spatial and temporal expression of starch biosynthesis genes. This network involves multiple transcription factor (TF) families, including MYB (ZmMYB14, ZmMYB138, and ZmMYB115) [1, 17], NAC (ZmNAC36, ZmNAC128, ZmNAC130, and ZmNAC126) [18–20], and bZIP (ZmbZIP91 and ZmbZIP22) [21–23], together with key regulators such as Opaque2 (O2) and PBF [24, 25]. These TFs directly regulate pathway genes—O2 mainly targets SuSy coding genes, whereas AGPase subunits and other biosynthetic components are controlled by TFs such as ZmbZIP22, ZmNAC128/130, and ZmMADS1a [19, 23, 26]. ZmbZIP22 acts as a master regulator coordinating most starch-synthesis-related genes [23]. TF activity is further modulated by sucrose availability, plant hormones, and epigenetic modifications, enabling precise control of starch deposition [1].

Despite these advances in defining regulatory components, a major translational gap remains between genetic discovery and breeding application. Genome-wide association studies (GWAS) and linkage mapping have identified numerous quantitative trait loci (QTLs) and candidate regulators associated with SC [27–29]; however, most starch-biosynthesis–related TFs have been characterised through reverse genetics in a limited number of genetic backgrounds. As a result, systematic functional validation of TFs in breeding-relevant populations—and evaluation of their haplotype effects—remain key bottlenecks. Bridging this gap will require an integrated strategy combining population genetics, molecular biology, and functional genomics to translate genomic signals into practical breeding tools.

In this study, we employed an integrated framework to identify starch-associated TFs with breeding potential in maize. Using multi-environment phenotyping, GWAS, and transcriptome profiling of subpopulations contrasting in SC, we found that coordinated expression of starch biosynthesis genes underlie SC variation. We then prioritised TF candidates by integrating GWAS signals with transcriptome correlation analysis, identifying *ZmMYB71*,* ZmMYB4*, and *ZmGNAT16* as strong candidates that show expression divergence and are closely associated with multiple starch biosynthesis genes. Haplotype analysis further supported their breeding relevance. Functional characterisation demonstrated that *ZmMYB71* negatively regulates starch accumulation by modulating the expression of core starch biosynthesis genes and associated enzymatic activities. Collectively, these findings provide mechanistic insight into the transcriptional control of kernel starch deposition and provide genetic resources to support breeding of maize lines with improved starch content.

## Results

### Variation in kernel SC and phenotypic characterisation of subpopulations

In the present study, a maize diversity panel comprising 341 inbred lines collected from the northwestern and Huang-Huai-Hai regions of China was evaluated. This panel has been used previously for GWAS of traits such as kernel protein content and disease resistance [30]. Kernel SC was measured over two consecutive years at two locations, Changping, Beijing (2018CP and 2019CP) and Gongzhuling, Jilin (2018GZL and 2019GZL), China. Across environments, kernel SC showed substantial phenotypic variation, ranging from 56.33% to 75.35%, and exhibited significant correlations among environments (*r* = 0.27–0.56, *P* < 0.01) (Fig. S1, Table S1), suggesting a considerable genetic contribution to SC variation.

**Fig. 1:**
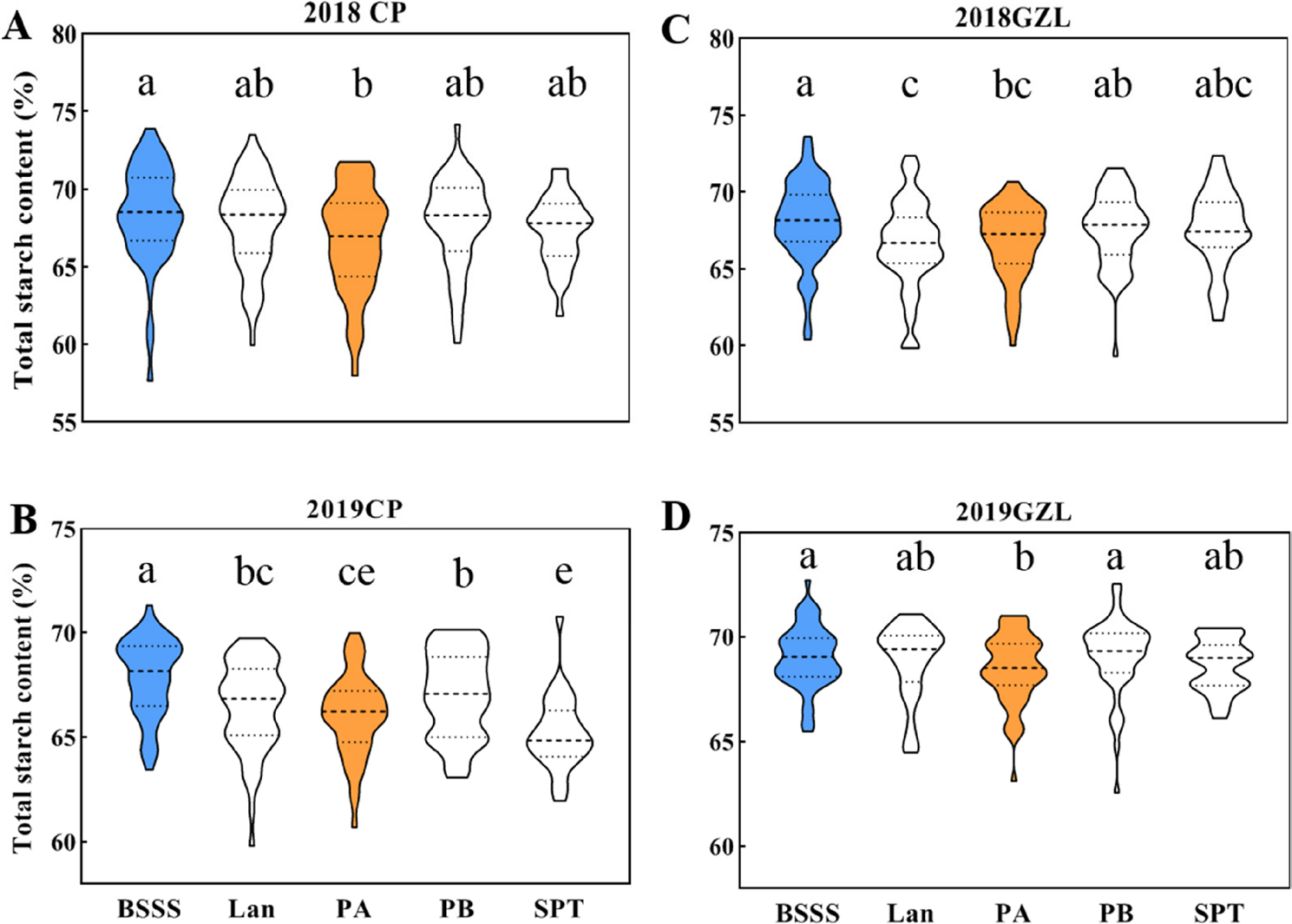
Multiple comparisons of kernel starch content (SC) among maize subpopulations across environments. (**A**–**B**) Changping, Beijing (2018CP and 2019CP). (**C**–**D**) Gongzhuling, Jilin (2018GZL and 2019GZL). BSSS, Iowa Stiff Stalk Synthetic; Lan, Lancaster Surecrop; PA, Partner A; PB, Partner B; SPT, Tangsipingtou. Different letters above violin plots indicate significant differences among subpopulations within the same environment (one-way ANOVA followed by multiple comparisons, *P* < 0.05)

The inbred lines were classified into five subpopulations: Iowa Stiff Stalk Synthetic (BSSS), Lancaster Surecrop (Lan), Partner A (PA), Partner B (PB), and Tangsipingtou (SPT; a Chinese landrace and its derivatives) following the previous study [30]. To evaluate SC variation among subpopulations, one-way ANOVA followed by multiple comparisons was performed within each environment (Fig. 1). In 2018CP, the BSSS subpopulation showed significantly higher SC than the PA subpopulation. In contrast, the SC of Lan, PB, and SPT subpopulations did not differ significantly from those of other subpopulations. In 2018GZL, the SC of the BSSS subpopulation was higher than those of the Lan and PA subpopulations. The SC was higher in PB than that in Lan, whereas the contents in the SPT subpopulation did not differ from those of other subpopulations. In 2019CP, BSSS exhibited the highest SC among all subpopulations, with higher contents in PB than those in PA and SPT, whereas SC of Lan exceeded that of SPT. In the 2019GZL, SC in BSSS and PB subpopulations were comparable; both significantly higher than that of PA (the lowest SC). Furthermore, SC in PA, Lan, and SPT showed no significant difference. Overall, the BSSS subpopulation consistently showed relatively high kernel SC, with average values ranging from 67.90% to 69.02%, whereas PA tended to show lower SC (66.06%–68.46%). The difference between SC of these two subpopulations across environments (0.60%-1.84%, with 0.82%–2.79% pensentage change) was significant (*P* = 2.9 × 10^− 5^–0.048), which was consistent with the comparisons based on BLUE values across subpopulations (Table S1).

### Identification of loci and genes associated with kernel SC through GWAS analysis

**Fig. 2:**
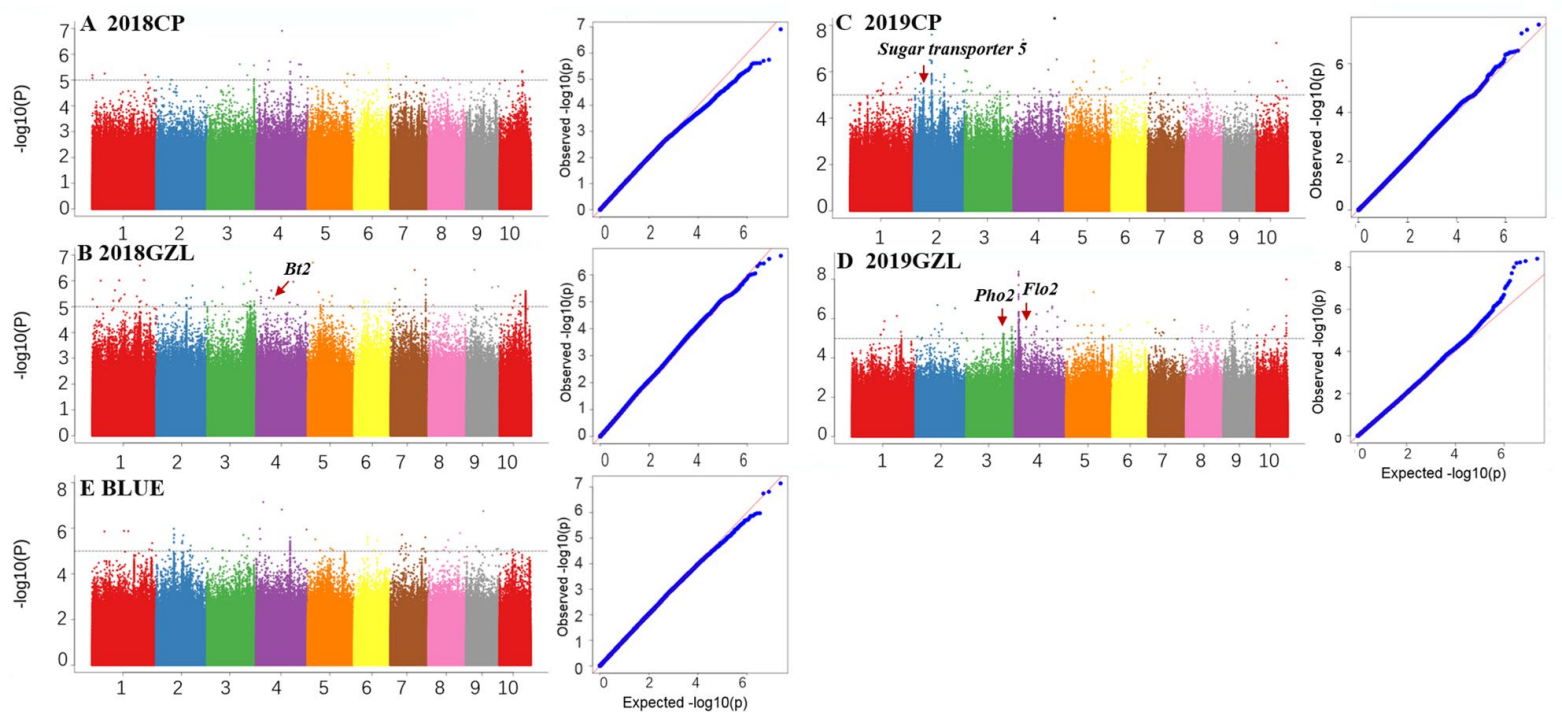
Genome-wide association study (GWAS) of kernel starch content. Manhattan plots for 2018CP (**A**), 2018GZL (**B**), 2019CP (**C**), 2019GZL (**D**), and the BLUE value (**E**) across environments. The x-axis represents maize chromosomes 1–10

Using whole-genome resequencing data, we identified 11,929,554 high-quality single-nucleotide polymorphisms (SNPs; −log_10_(*P*) = 5) for GWAS, of which 515 SNPs showed significant association with kernel SC. Specifically, we identified 32, 152, 121, 183, and 78 significant SNPs in 2018CP, 2018GZL, 2019CP, 2019GZL, and the starch BLUE value, respectively (Fig. 2, Table S2). The highest −log_10_(*P*) values were 6.91, 6.71, 7.60, 8.39, and 7.14. Nine of these SNPs overlapped with previously reported QTL intervals (or LD regions of QTL) associated with kernel SC (Table S3) [28, 29].

Subsequently, following the procedure outlined by Han et al. [31], we identified 348 putative genes in the supporting intervals, which were primarily enriched in pathways associated with starch and sugar metabolism, circadian rhythm, RNA degradation, and other pathways (Fig. S2). Among these, four key genes—*Zm00001d050032*,* Zm00001d049243*, *Zm00001d042842*, and *Zm00001d00347*—have been annotated previously to be strongly associated with maize kernel SC. *Zm00001d050032*, detected in 2018GZL, encodes brittle endosperm2 (*Bt2*). *Zm00001d049243* and *Zm00001d042842*, detected in 2019GZL, encode floury endosperm2 (*Flo2*) and plastidial starch phosphorylase 2 (*Pho2*), respectively, both of which participate in starch biosynthesis [32–34]. In addition, *Zm00001d003473*, detected in 2019CP, encodes a sugar transport protein. Homologs in this family mediate sucrose uptake into endosperm cells during grain filling and are critical for kernel starch accumulation [2].

### Prioritisation of key candidate genes through analysis of starch biosynthesis gene expression across maize subpopulations

**Fig. 3:**
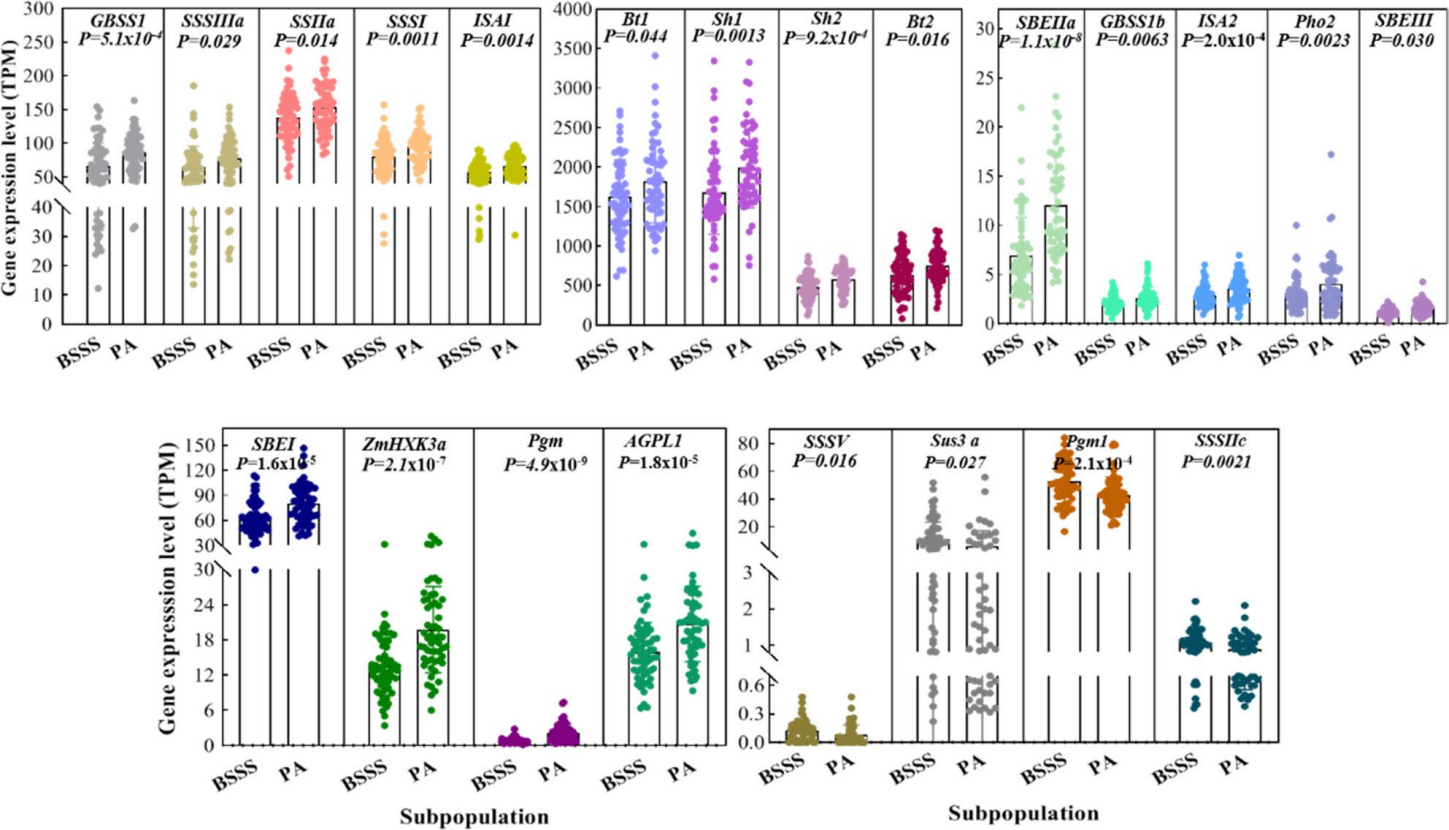
Differential expression of starch biosynthesis genes between the high-starch BSSS and low-starch PA subpopulations. Statistical significance was determined using a two-sided Student’s t-test (*P* values shown)

Previous transcriptome profiling of the elite B73 inbred line has shown that starch biosynthesis genes are highly expressed in the endosperm from 6 to 36 days after pollination (DAP), with specific peak expression windows for different genes [35]. The 16–21 DAP represents a key developmental period for studying transcriptional regulation of starch biosynthesis [24, 26, 36, 37]. Based on this rationale, endosperm samples collected at 20 DAP from 61 maize inbred lines of the high-SC BSSS subpopulation and 58 lines from the low-SC PA subpopulation were subjected to transcriptome sequencing (Tables S4 and S5). A total of 38 known starch biosynthesis genes encoding key enzymes, including SuSy, UGPase, PGM, AGPase, GBSS, SSS, SBE, DBE, DPE, and PHO, were compared between BSSS and PA subpopulations. The expression levels of *Sus3a*, *Pgm1*, *SSIIc*, and *SSSV* were 1.74-, 1.24-, 1.23-, and 1.55-fold higher, respectively, in BSSS than those in PA. Conversely, 18 genes, including *GBSSI*, *SSIIa*, *SSIIIa*, *SS1*, and *ISA1*, showed reduced expression in BSSS, ranging from 0.41- to 0.90-fold relative to PA (Fig. 3; Table S6). Overall, 58% of the analysed starch biosynthesis genes were significantly differentially expressed between the two subpopulations. These differentially expressed genes (DEGs) included those preferentially expressed in embryo/seed tissues and those enriched in endosperm/seed tissues, and they primarily encode enzymes involved in sucrose conversion and starch biosynthesis (SuSy, AGPase, GBSS, SSS, SBE, and DBE). Collectively, these findings suggest that coordinated transcriptional differences in starch biosynthesis genes may contribute to the observed subpopulation-level variation in kernel SC.

**Fig. 4:**
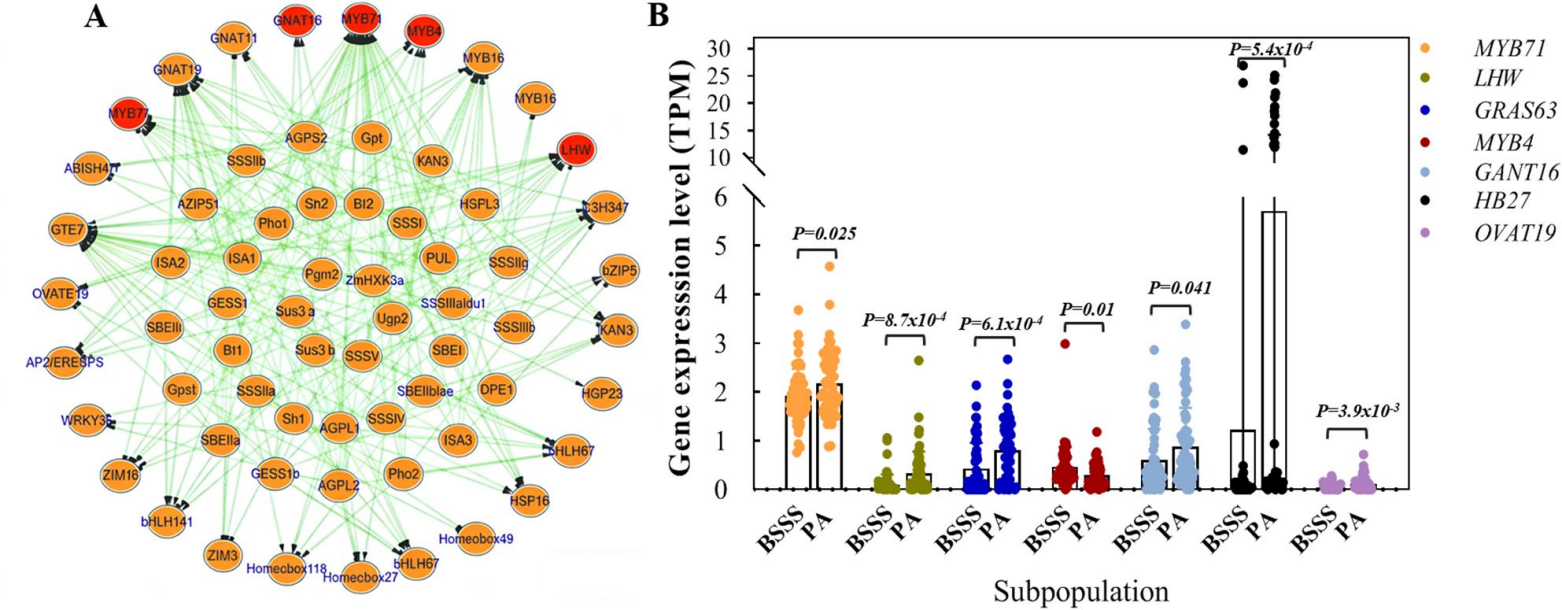
Transcription factors associated with the expression of starch biosynthesis genes in 20-DAP endosperm. (**A**) Correlation analysis between transcription factors and starch biosynthesis genes based on endosperm transcriptomes. (**B**) Seven transcription factors showing significant differential expression between BSSS and PA subpopulations. Statistical significance was determined using a two-sided Student’s t-test, as indicated by the *P* values

Transcription factors often exert pleiotropic effects; thus, variation in TF expression may lead to coordinated changes in downstream starch biosynthesis genes. Among GWAS candidate genes, 29 TFs were identified, including MYB and MYB-related, bHLH, Homeobox, GNAT, and HSF family members (Table S7). To prioritise key regulatory TFs associated with SC variation, correlations between the expression of these 29 TFs and 38 known starch biosynthesis genes were estimated using 20 DAP endosperm transcriptome data. Seven TFs (*ZmMYB71*, *ZmGTE7*, *ZmGRAS63*, *ZmLHW*, *ZmFHA10*, *ZmC3H347*, and *ZmMYB77*) showed significant correlations (*r* = 0.18–0.70; *P* < 0.05) with more than 10 starch biosynthesis genes (Fig. 4A, Table S8). Specifically, *ZmMYB71* showed association with 32 genes, which included 20 DEGs between BSSS and PA subpopulations. *ZmGTE7* was correlated with 22 genes (16 DEGs), *ZmGRAS63* with 18 genes (15 DEGs), and *ZmLHW* with 12 genes (11 DEGs). *ZmFHA10*, *ZmC3H347*, and *ZmMYB77* were correlated with 11–14 genes each, including 5–8 genes showing subpopulation-level differences.

Further analysis identified seven TFs—*ZmMYB71* (*Zm00001d026362*), *ZmLHW* (*Zm00001d004868*), *ZmGRAS63* (*Zm00001d022034*), *ZmMYB4* (*Zm00001d048647*), *ZmGNAT16* (*Zm00001d044368*), *ZmHB27* (*Zm00001d015671*), and *ZmOVATE19* (*Zm00001d044167*)—that were significantly differentially expressed between BSSS and PA subpopulations and were correlated with 3–32 starch biosynthesis genes (Fig. 4B). These TFs were therefore considered strong candidates underlying natural variation in kernel SC.

### Co-expression analysis of haplotype, transcript levels, and SC in candidate TFs

**Fig. 5:**
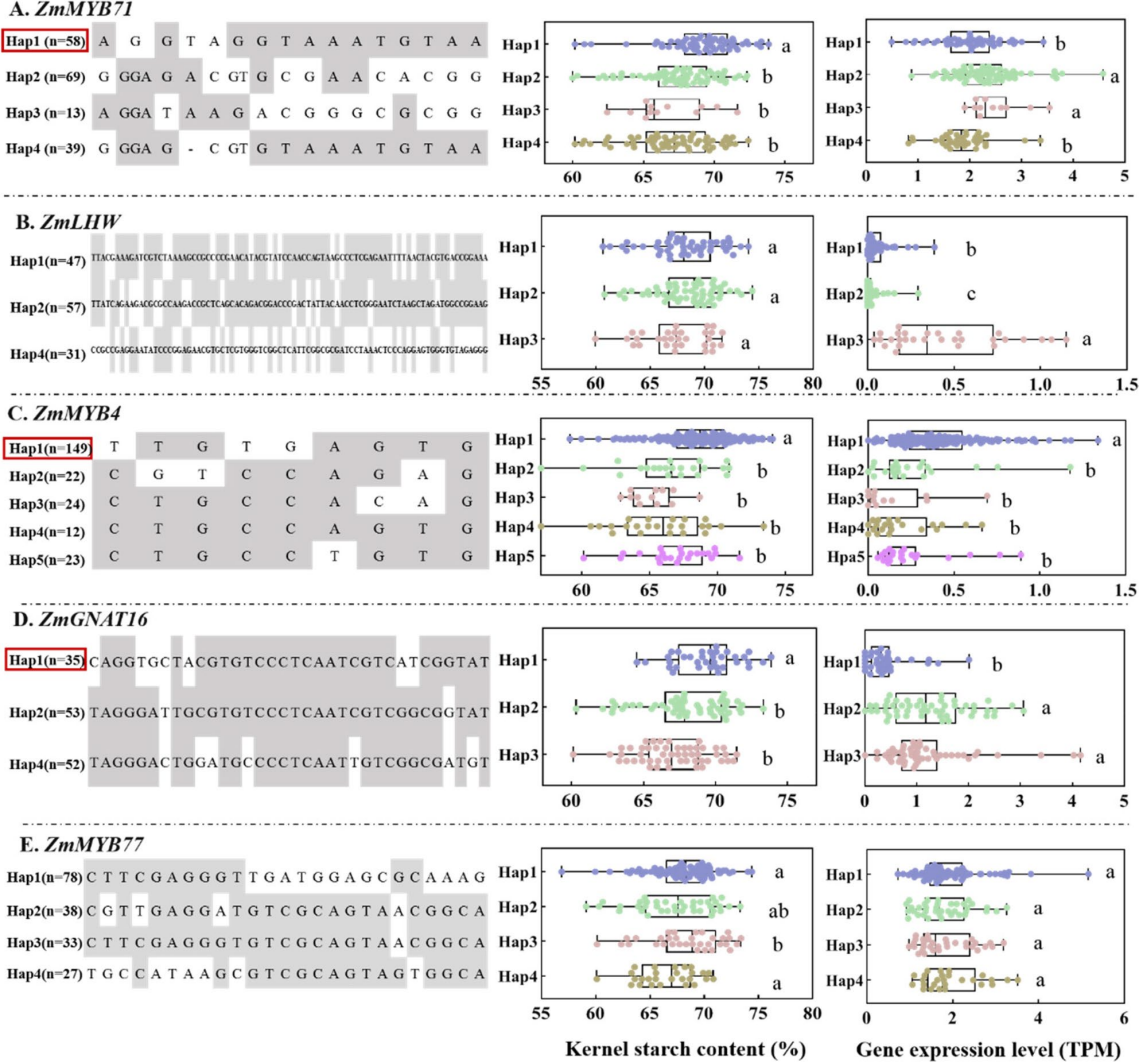
Kernel starch content and endosperm expression levels of candidate transcription factors across haplotypes. Haplotype-based comparisons for (**A**) *ZmMYB71*, (**B**) *ZmLHW*, (**C**) *ZmMYB4*, (**D**) *ZmGNAT16*, and (**E**) *ZmMYB77*. Different letters above the plots indicate significant differences among haplotypes in BLUE values of kernel SC one-way ANOVA (*P* < 0.05)

**Fig. 6:**
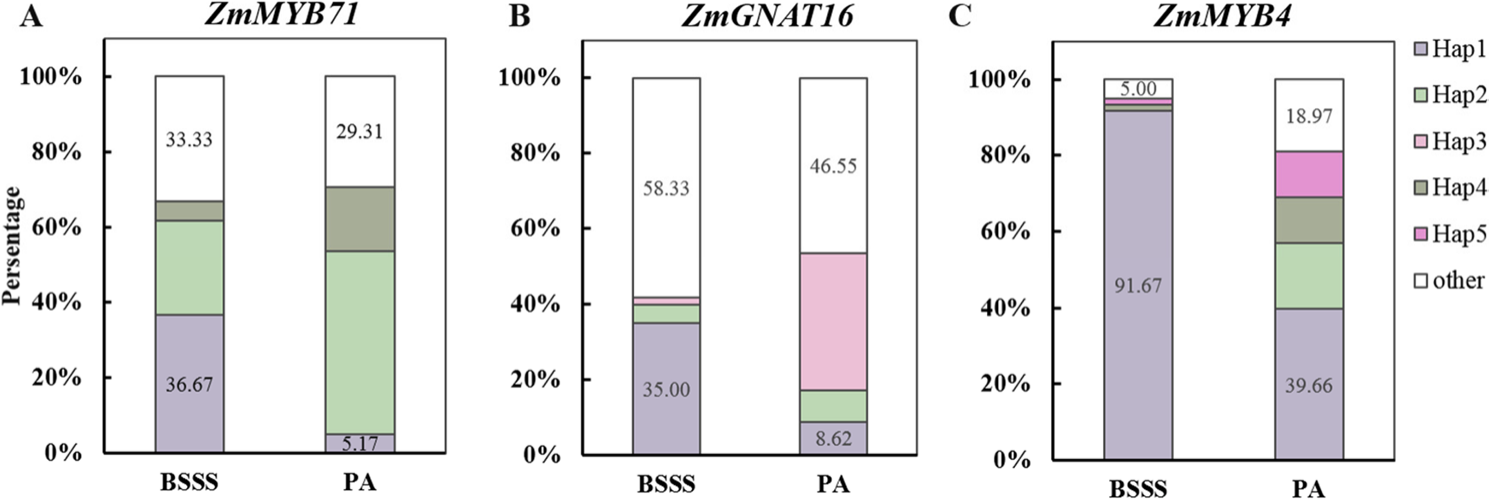
Distribution of favourable haplotypes of candidate transcription factors in BSSS and PA subpopulations. Haplotype frequencies of (**A**) *ZmMYB71*, (**B**) *ZmGNAT16*, and (**C**) *ZmMYB4*

Based on the analyses above, *ZmMYB71*, *ZmLHW*, *ZmMYB4*, *ZmGNAT16*, and *ZmMYB77* were selected for haplotype analysis. Using SNPs in promoter and coding regions, 3–5 major haplotypes were identified for each gene (Fig. 5A–E). For *ZmMYB71*, Hap1 showed the highest kernel SC (69.04%), which was 1.64%–2.66% higher than that for the other haplotypes. No significant differences in kernel SC were observed among *ZmLHW* haplotypes. For *ZmMYB4* and *ZmGNAT16*, Hap1 conferred 1.63%–3.30% and 1.51%–2.38% higher kernel SC, respectively, than those for the other haplotypes. For *ZmMYB77*, Hap1 and Hap2 exhibited significantly higher SC than Hap3, but did not differ significantly from Hap4. Haplotype-based expression analysis using 20-DAP endosperm transcriptome data revealed that *ZmMYB71* expression in Hap1 was significantly lower than those in Hap2 and Hap3, whereas *ZmMYB4* expression in Hap1 was significantly higher than those in the other haplotypes. *ZmGNAT16* expression in Hap1 was lower than those in Hap2 and Hap3. Although *ZmLHW* haplotypes did not differ significantly in SC, their transcript levels differed significantly. Moreover, *ZmMYB77* showed no clear haplotype-dependent differences in expression. Together, these findings suggest that favourable alleles of *ZmMYB71*, *ZmMYB4*, and *ZmGNAT16* contribute to increased kernel SC in maize. The expression patterns further suggest that *ZmMYB71* and *ZmGNAT16* may act as negative regulators of kernel SC, whereas *ZmMYB4* may act as a positive regulator. In addition, Hap1 frequencies for *ZmMYB71*, *ZmMYB4*, and *ZmGNAT16* were 36.67%, 91.67%, and 35.00% in the BSSS subpopulation, but only 5.17%, 39.66%, and 8.62% in PA, respectively (Fig. 6A–C). Analysis of published spatiotemporal transcriptome datasets further indicated that *ZmMYB71* is expressed at relatively high levels from 6 to 38 DAP, *ZmMYB4* expression increases beginning at 26 DAP [35], whereas *ZmGNAT16* exhibits consistently low expression throughout the filling stage (Fig. S3).

### Functional analysis of *ZmMYB71* in kernel SC regulation

**Fig. 7:**
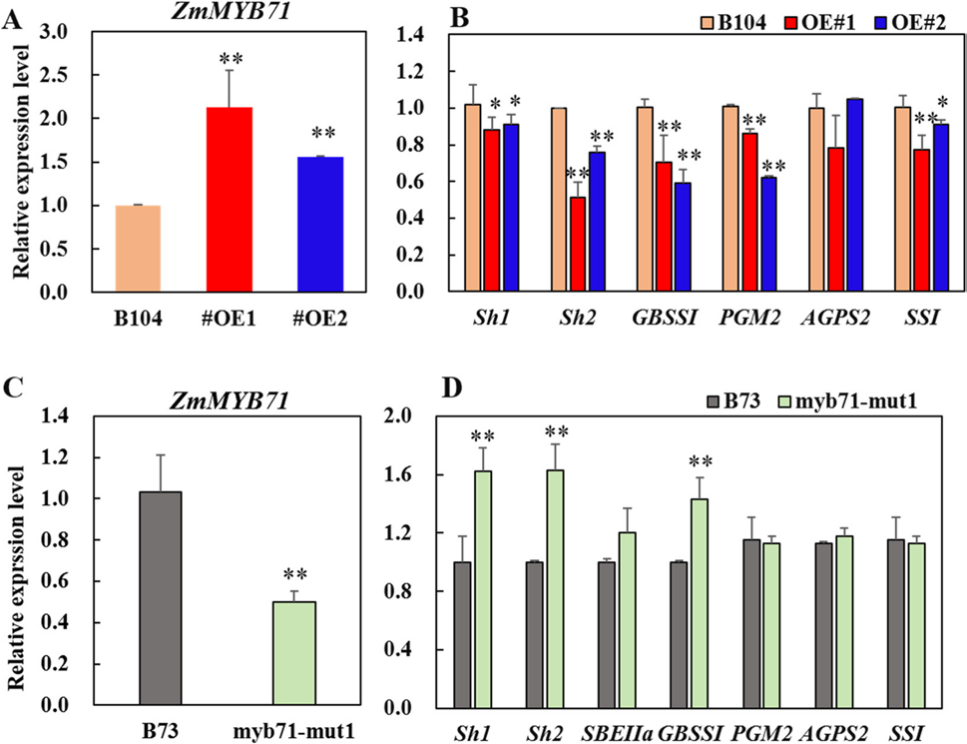
Expression analysis of *ZmMYB71* and starch biosynthesis genes in overexpression and mutant lines. (**A**, **C**) *ZmMYB71* transcript levels at 20-DAP endosperm. (**B**, **D**) Expression levels of starch biosynthesis genes in (**B**) overexpression lines and (**D**) mutant lines compared with their corresponding wild-type controls. Bars represent mean ± SE of three biological replicates. Statistical significance was determined using two-sided Student’s t-test, with ^*^ *P* < 0.05, ^**^ *P* < 0.01

Building on previous work [31], we confirmed that *ZmMYB71* represses specific starch biosynthesis genes (*Sh1*, *Sh2*, *GBSSI*, and *SBEIIa*) by binding to GATATC/TTAGGG cis-elements, as shown by dual-luciferase and yeast one-hybrid assays. To further elucidate the role of ZmMYB71 in SC regulation, we analysed two independent overexpression lines (*ZmMYB71-OE#1* and *ZmMYB71-OE#2*) and two loss-of-function mutants (*myb71-mut1* and *myb71-mut2*). In 20-DAP endosperm tissues from the overexpression lines, which showed significantly elevated *ZmMYB71* transcript levels, multiple starch biosynthesis genes—including *Sh1*, *Sh2*, *GBSSI*, *PGM2*, and *SSI*—were markedly downregulated (Fig. 7A). Conversely, these genes were upregulated in the *myb71-mut1* mutant, in which *ZmMYB71* expression was significantly reduced (Fig. 7B).

**Fig. 8:**
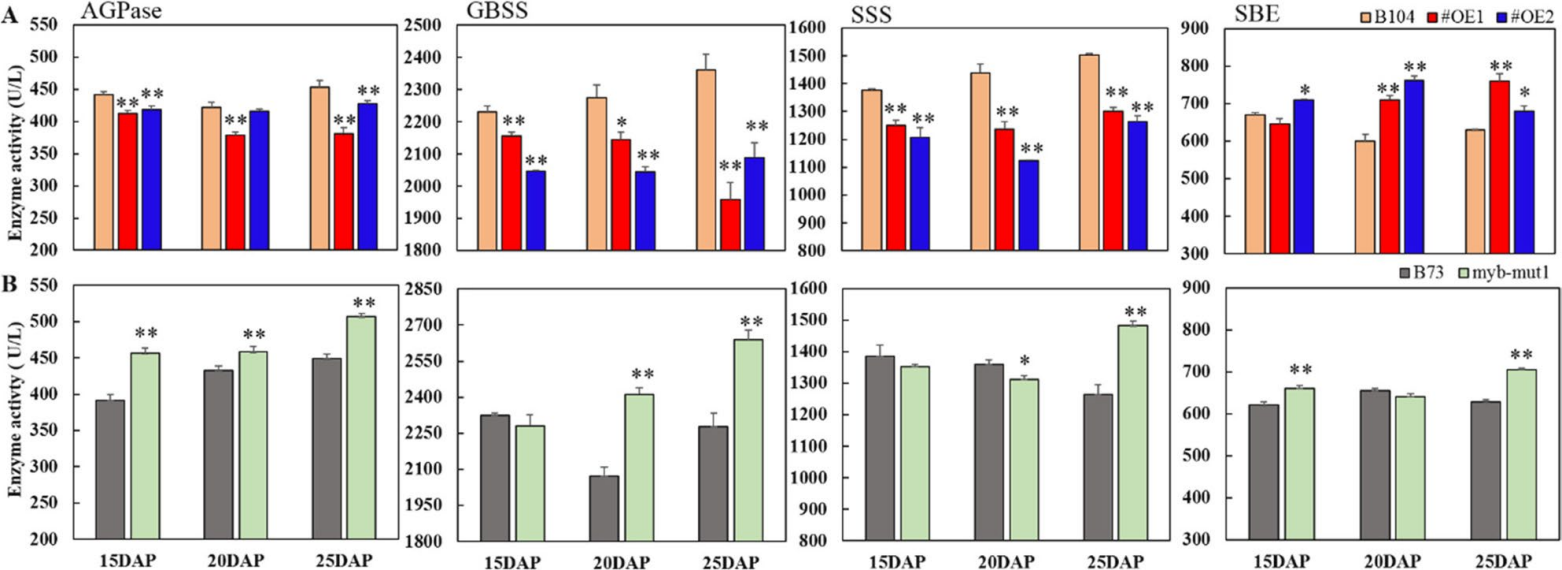
Starch biosynthesis enzyme activities in *ZmMYB71* overexpression and mutant lines. (**A**) *ZmMYB71*-overexpressing lines (OE) and (**B**) *zmmyb71* mutant line (mut1). Each bar represents the mean ± SE of three biological replicates. Statistical significance was determined using two-sided Student’s t-test, with ^*^ *P* < 0.05, ^**^ *P* < 0.01

Consistent with the transcriptional changes, starch biosynthetic enzyme activities were also altered. In the *ZmMYB71* overexpression lines, AGPase, GBSS, and SSS activities were significantly decreased in endosperm between 15 and 25 DAP (Fig. 8A). Specifically, *ZmMYB71-OE#1* showed decreases of 3.26%–16.06%, while *ZmMYB71-OE#2* showed decreases of 5.17%–21.93%. In contrast, SBE activity increased significantly in *ZmMYB71-OE#1* by 18.15% and 21.39% at 20 and 25 DAP, respectively, and by 26.71% and 7.73% in *ZmMYB71-OE#2*, respectively. In the *myb71-mut1* mutant, AGPase activity increased by 16.48%, 5.79%, and 12.85% at 15, 20, and 25 DAP, respectively, while GBSS activity increased by 16.36% and 15.87% at 20 and 25 DAP, respectively. Although SSS activity decreased at 20 DAP, it increased by 17.20% at 25 DAP. SBE activity increased by 6.58% at 15 DAP, remained stable at 20 DAP, and increased by 12.36% at 25 DAP (Fig. 8B).

**Fig. 9:**
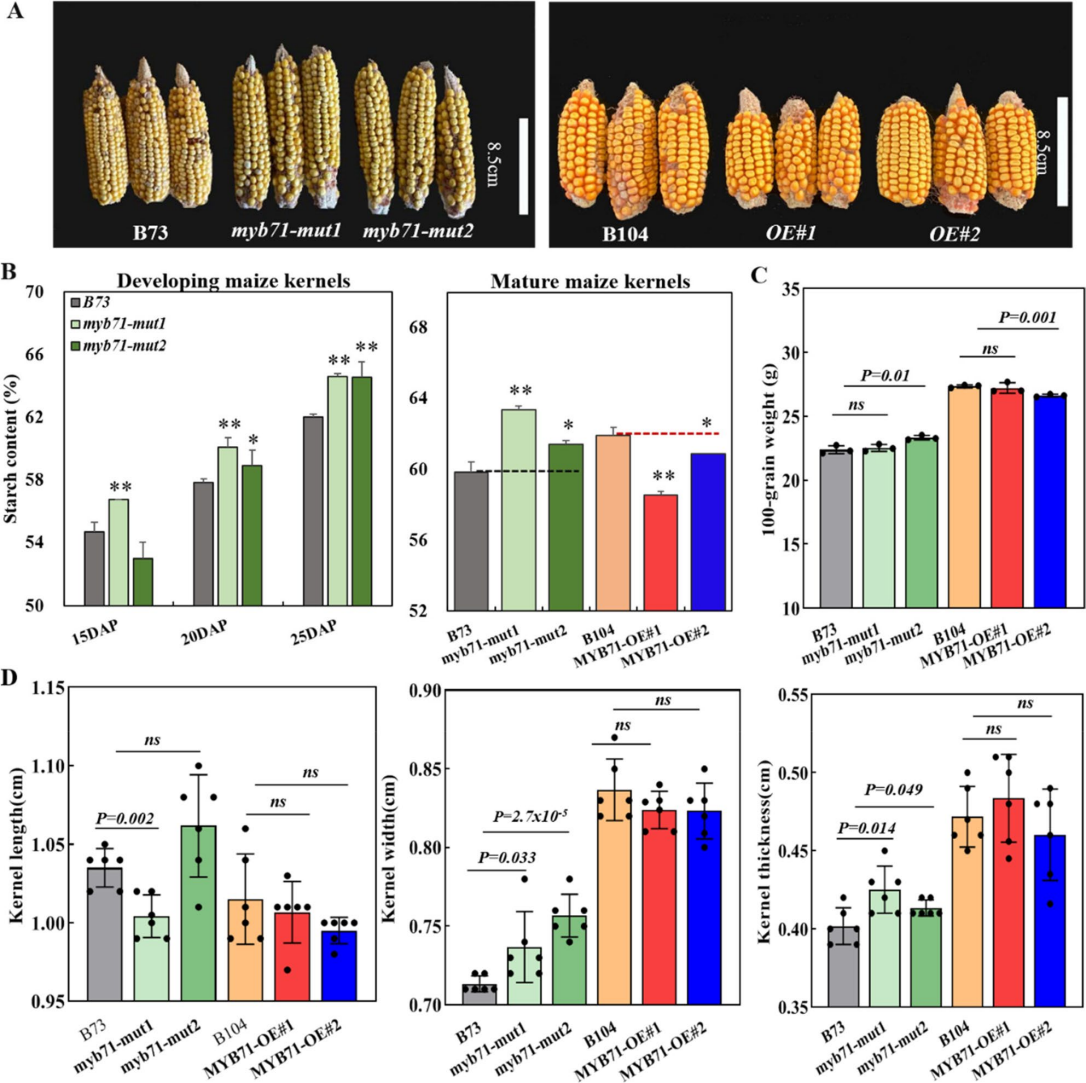
Phenotypic characterisation of *ZmMYB71* overexpression and mutant lines. (**A**) Representative mature ears of homozygous lines. (**B**) Starch content in developing kernels and mature kernels. (**C**) 100-kernel weight. (**D**) Kernel length, width, and thickness. In (**B**–**C**), bars represent mean ± SE of three biological replicates. Statistical significance was determined using two-sided Student’s t-test, with * P < 0.05, ** P < 0.01; ns, no significant differences

**Fig. 10:**
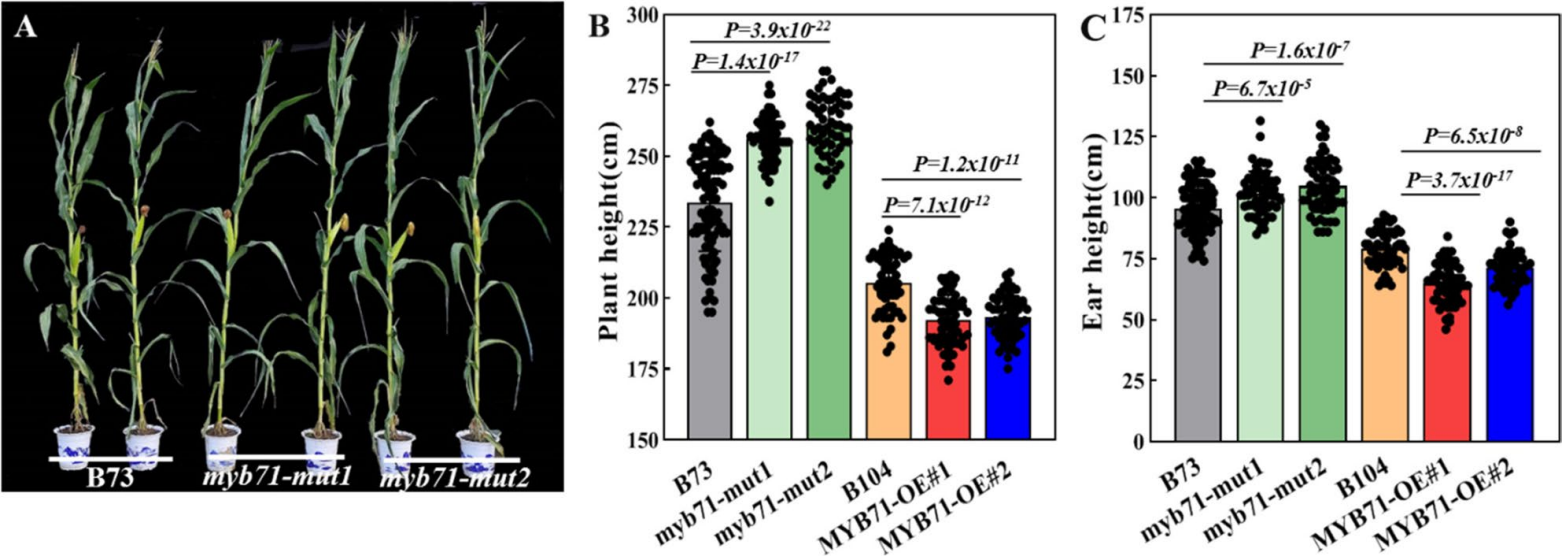
Plant architecture traits in *ZmMYB71* overexpression and mutant lines. (**A**) Representative adult plants of myb71 mutant lines. (**B**) Plant height and (**C**) ear height of ZmMYB71 overexpression and mutant lines. Statistical significance was determined using two-sided Student’s t-test, as indicated by the *P* values

Kernel SC in *myb71-mut1* differed significantly from that in the B73 control with 3.72%, 3.83%, and 4.19% higher contents at 15, 20, and 25 DAP, respectively. In addition, *myb71-mut2* showed a significant increase by 4.09% at 25 DAP (Fig. 9A, B). In open-pollinated ears, kernel SC increased from 59.84% in B73 to 63.38% and 61.44% in *myb71-mut1* and *myb71-mut2*, respectively, consistent with results from self-pollinated ears reported previously [31]. In contrast, *ZmMYB71-OE#1* (58.88%) and *ZmMYB71-OE#2* (60.87%) lines exhibited a decrease in kernel SC compared with the B104 control (61.92%). In addition, 100-kernel weight increased in *myb71* mutants. In contrast, a decreasing trend was observed in the overexpression lines (Fig. 9C). Consistently, both mutants showed significant increases in kernel length, width, and thickness, except for kernel length in *myb71-mut2*. In contrast, the overexpression lines showed decreasing trends in grain dimensions without statistical significance (Fig. 9D). Moreover, *ZmMYB71* overexpression reduced plant height and ear position significantly relative to B104. In contrast, the *myb71* mutants showed increased plant height and ear position relative to B73 (Fig. 10A–C). These phenotypic differences may be partly attributable to elevated *ZmMYB71* expression in stem tissue, which may also indirectly influence starch accumulation in kernels.

## Discussion

Maize, a model organism for quantitative genetics, has been extensively investigated using GWAS and QTL mapping to identify genes associated with kernel SC. Luo et al. narrowed the GWAS-associated gene list to 248 candidates by integrating transcriptomic differences between two maize lines (ZNC442 and SCML0849) that exhibit distinct starch granule characteristics [38]. Similarly, RNA-sequencing (RNA-seq) of near-isogenic lines at the qHS3 locus revealed 76 genes harbouring nonsynonymous SNPs and 384 DEGs within the introgressed interval, providing valuable targets for kernel SC improvement [39]. Hu et al. further demonstrated that prioritising genes involved in carbohydrate metabolism enabled the successful cloning and characterisation of *ZmTPS9*, which contributes to natural variation in kernel SC [29]. Although numerous QTLs and candidate genes have been reported [28, 36, 40], their translational value for breeding remains uncertain.

In the present study, we selected the BSSS and PA inbred subpopulations as contrasting models because they displayed significant and environmentally stable differences in kernel SC (Fig. 1). These two subpopulations had comparable sample sizes and represented phenotypic extremes (Table S1), thereby supporting robust differential expression analysis. We identified 22 starch biosynthesis genes that were differentially expressed between BSSS and PA endosperms at 20 DAP (Fig. 3; Table S6), suggesting that upstream regulatory factors—particularly TFs—may play key roles in driving subpopulation-level differences in kernel SC.

Core starch biosynthesis genes (e.g., *Sh1*, *Bt2*, *Sh2*, and *SSI*) exhibit distinct spatiotemporal expression patterns, with high expression typically observed from 10 to 32 DAP and sustained expression up to 38 DAP [35]. Regulatory TFs often show coordinated expression with starch biosynthetic genes, consistent with transcriptional co-regulation. For example, in rice, *RSR1* is co-expressed with starch biosynthesis genes, and mutation of *RSR1* alters the expression of many starch biosynthesis-related genes [41]. In maize, *ZmaNAC36* is co-expressed with genes encoding AGPase subunits, SSI, GBSSIb, SBEI, and others [18]. Several additional TFs have been implicated in kernel SC regulation through co-expression analyses, including ZmABI4 [42], ZmEREB156 [43], ZmbZIP91 [21], ZmNAC128/130 [19], and ZmNAC126 [20]. Although many TFs originate from pathways not directly involved in starch metabolism, several TF loci have been mapped to QTL regions significantly associated with kernel SC [27, 29, 31, 36, 40]; however, functional validation of favourable alleles remains limited.

In this study, we integrated GWAS-derived TF candidates with transcriptomic data-based correlation and haplotype analyses, prioritising ZmMYB71, ZmMYB4, and ZmGNAT16 as candidate regulators of kernel SC. These TFs were significantly differentially expressed between the BSSS and PA subpopulations, and their favourable haplotypes were enriched in the high-starch BSSS subpopulation (Figs. 5, 6 and 7). Co-expression analysis based on transcriptomic data from 53 B73 seed samples (embryos, endosperms, and whole seeds) further supported their association with starch biosynthesis genes (Fig. S4). This result was consistent with the 20-DAP endosperm dataset from 119 inbred lines, in which *ZmMYB71*, *ZmMYB4*, and *ZmGNAT16* were correlated with the expression of 32, 7, and 3 starch biosynthesis genes, respectively (Table S8).

The MYB TF family plays diverse roles in plant development, nutrient accumulation, and stress responses [44]. ZmMYB71 and ZmMYB4 belong to the MYB-related and MYB-like subfamilies, respectively. In other plant species, MYB TFs have been implicated in regulating the biosynthesis or accumulation of storage compounds. For example, in wheat, TaODORANT1 suppresses seed storage protein genes, and RNAi-mediated knockdown increases grain protein content and thousand-kernel weight without apparent effects on agronomic traits [45]. In Arabidopsis, AtMYB89 represses *WRINKLED1*, *LEC1-LIKE*, and multiple lipid biosynthesis genes [46], whereas mutations in *PHR1*, a MYB-related TF, impair AGPase activity and reduce sugar and starch accumulation [47]. In rice, mutations in GL4 (a MYB-like protein) affect grain size, and allele replacement improves yield [48]. Moreover, AtMYBL2 negatively regulates flavonoid and anthocyanin biosynthesis [49, 50]. Consistent with the regulatory roles of MYB TFs, our data indicate that ZmMYB71 acts as a transcriptional repressor (Fig. 5A). Overexpression of *ZmMYB71* reduced the expression of *Sh1*, *Sh2*, *GBSSI*, and other starch biosynthesis genes (Fig. 7B), resulting in decreased activity of key enzymes, including AGPase, SSS, and GBSS, as well as reduced kernel SC (Figs. 8 and 9). In contrast, *zmmyb71* mutants exhibited increased expression of starch biosynthesis genes and enhanced kernel SC. Unlike ZmMYB71, ZmMYB4 may promote starch synthesis by activating starch biosynthesis genes (Fig. 5C), consistent with the dual capacity of MYB family members to activate or repress downstream targets depending on context.

GNAT (GCN5-related N-acetyltransferase) proteins participate in diverse biological processes, including transcriptional regulation, stress responses, and metabolic control [51]. In mammals, p300/CBP-associated factor (a GNAT TF) acts as a transcriptional coactivator and promotes gene expression [52]. In this study, ZmGNAT16 is presumed to function as a transcriptional regulator affecting kernel SC (Fig. 5D); however, further functional validation will be required to clarify its regulatory role and downstream targets.

The use of diverse germplasm resources, including local and exotic accessions, has increased genetic diversity available for maize improvement. Pyramiding favourable alleles across multiple loci is a key breeding strategy to enhance complex agronomic traits. In this study, the number of favourable alleles at *ZmMYB71*, *ZmMYB4*, and *ZmGNAT16* varied substantially among inbred lines (Table S9). In the BSSS subpopulation, 1.67%, 46.67%, 38.33%, and 13.33% of lines carried 0, 1, 2, or 3 favourable alleles, respectively, whereas in PA the corresponding frequencies were 60.34%, 29.31%, 6.90%, and 3.45%, respectively. Consistently, analysis of 109 inbred lines showed that lines carrying two or three favourable alleles displayed significantly higher SC (69.38% and 70.98%, respectively) than those carrying one or no favourable alleles (67.02% and 66.74%, respectively; *P* < 0.001), supporting additive effects of these alleles on kernel SC (Fig. S5). The BSSS subpopulation, represented by lines such as B73, Tie7922, and PH6WC, and the PA subpopulation, represented by Ye478 and Zheng58, constitute important genetic resources for elite maize breeding in China. Therefore, pyramiding favourable haplotypes of *ZmMYB71*, *ZmMYB4*, and *ZmGNAT16* may provide useful selection criteria for developing high-SC germplasm.

Overall, the study identified *ZmMYB71*,* ZmMYB4*, and *ZmGNAT16* as important regulators potentially contributing to natural variation in starch biosynthesis in maize kernels. However, further experimental validation is required to confirm the functions of *ZmMYB4* and *ZmGNAT16*, including evidence of their binding and regulatory effects on downstream starch biosynthesis genes.

## Conclusions

Based on phenotypic evaluation across multiple environments, this study identified the BSSS and PA subpopulations as groups with relatively high and low kernel starch content (SC), respectively. Transcriptome analysis of starch biosynthesis genes indicated that most genes showed coordinated differential expression between these two subpopulations, based on endosperm samples from 119 inbred lines. These findings suggest that TFs may contribute substantially to subpopulation-level differences in kernel SC. By integrating GWAS signals with haplotype and transcriptome expression analyses, we prioritised ZmMYB71, ZmMYB4, and ZmGNAT16 as key TF candidates associated with kernel SC variation. Functional analyses further demonstrated that both overexpression and mutation of *ZmMYB71* led to significant changes in kernel SC. Moreover, inbred lines carrying favourable haplotypes of these three TFs exhibited increased kernel SC. Collectively, these findings provide valuable genetic targets for breeding strategies aimed at improving starch content in maize.

## Materials and methods

### Plant materials and field experiments

A total of 341 maize inbred lines were developed and maintained by the Maize High-Quality and Stress-Resistant Genetics Group, Institute of Crop Sciences, Chinese Academy of Agricultural Sciences (CAAS). The inbred panel was evaluated during the summers of 2018 and 2019 at two locations in China: Changping, Beijing (2018CP and 2019CP), and Gongzhuling, Jilin Province (2018GZL and 2019GZL). Field density was maintained at 60,000 plants/ha. Five ears per replicate were manually self-pollinated, and mature kernels from the central region of each ear were collected for total kernel SC determination. Meteorological data were obtained from the national meteorological station. In Changping, the number of high-temperature days (> 35 °C) was lower in 2019 than in 2018, accompanied by reduced average temperatures in June and lower total precipitation. In Gongzhuling, mean temperatures from June to September 2019 were 1–5 °C lower than those in 2018. Although total annual precipitation was similar between years, rainfall in 2019 was mainly concentrated in August.

For functional validation, *zmmyb71* mutants (myb71-mut1 and myb71-mut2) were obtained from the maize ethyl methanesulfonate (EMS) mutant library (http://www.elabcaas.cn/memd/). These mutants harbor premature stop codon (CGA to TGA) in the 13th exon of the gene. and homozygous individuals were identified using PCR-based genotyping. For overexpression assays, the full-length coding sequence of *ZmMYB71* from B73 was cloned into the CUB vector under the control of the maize ubiquitin promoter to generate independent transgenic lines (*MYB71-OE#1* and *MYB71-OE#2*). Mutant and overexpression lines in the B73 and B104 backgrounds, respectively, were grown in Changping, Beijing, during the summers of 2023 and 2024 in four-row plots with three biological replicates. Pollination dates were recorded, and 1 DAP was defined as 24 h after self-pollination. For molecular analyses, endosperm tissues were dissected from kernels in the central region of ears at 15, 20, and 25 DAP (three ears per replicate) using sterile tweezers. Samples were immediately frozen in liquid nitrogen and stored at − 80 °C until further analysis.

### Kernel starch content evaluation

For the maize inbred panel, mature kernels were ground into a fine powder, dried to constant weight, and analysed for SC using the GB 5009.9–2016 method as previously described [31].

For developmental SC profiling in mutants and overexpression lines, SC was measured using an enzymatic digestion method. Briefly, approximately 50 mg of finely ground kernel powder was washed with 80% (v/v) ethanol to remove soluble sugars. After centrifugation, the pellet was resuspended in 2 mol/L KOH in sodium acetate buffer (pH 3.8) and vortexed for 30 s. Subsequently, thermostable α-amylase was added, and the suspension was incubated at 95 °C for 5 min to gelatinise starch. After cooling to 50 °C, 0.1 mL amyloglucosidase (3 U/mL) was added, and the mixture was vortexed and incubated at 50 °C for 30 min to achieve complete hydrolysis. The hydrolysate was diluted 10-fold with distilled water. For colourimetric quantification, 50 µL of diluted hydrolysate was mixed with 1.5 mL GOPOD reagent and incubated at 50 °C for 20 min. Absorbance was measured at 510 nm using a spectrophotometer (Multiskan GO, Thermo Fisher Scientific, USA). SC was calculated using a glucose standard curve (0–100 µg/mL). A conversion factor of 0.9 was applied to account for the molecular weight difference between glucose and starch polymers.

### RNA sequencing of maize endosperm

For transcriptome analysis, maize inbred lines were planted in Changping in 2019, and endosperms were collected at 20 DAP. Twenty immature kernels from two ears were pooled per replicate, flash-frozen in liquid nitrogen, and used for RNA extraction with TRIzol reagent (Thermo Fisher Scientific) following the manufacturer’s instructions. Library construction and sequencing were performed by Beijing Nuohezhiyuan Institute (Tianjin, China). Paired-end sequencing (150 bp) was conducted, generating an average of 7.7 Gb of high-quality reads per sample. Gene expression levels were quantified and normalised as transcripts per million (TPM).

### GWAS for kernel starch content measurement

GWAS was performed on kernel SC data using phenotypic data from the maize inbred lines and 11,929,554 high-quality SNPs. Candidate genes were defined as those located within ± 20 kb of significant SNP loci. The GWAS pipeline and analytical procedures were consistent with those described by Lu et al. [30].

### Correlation and co-expression analyses

To explore regulatory relationships between TFs and starch biosynthesis genes, TPM expression data from 119 inbred lines belonging to the BSSS and PA subpopulations were analysed (Tables S4 and S5). Pearson’s correlation coefficients were calculated for TF–target gene pairs (Table S8). Correlation analyses and *P*-value calculations were performed in R, and *P* < 0.05 was considered statistically significant. Dynamic TF–gene co-expression heatmaps were generated based on transcriptomic profiles of 53 dynamically expressed genes previously reported by Chen et al. [35]. Analyses and visualisation were performed using R packages including pacman, vegan, corrplot, and ggplot2.

### RNA extraction and quantitative PCR (qRT-PCR)

Total RNA was extracted from endosperm tissues collected at 20 DAP using the TransZol Up Plus RNA Kit (Tiangen, Beijing, China), following a previously described protocol [53]. RNA integrity was assessed using 1.5% agarose gel electrophoresis and NanoDrop spectrophotometry (A260/A280 > 1.8). First-strand cDNA was synthesised from 1 µg total RNA using the FastQuant RT Kit with gDNase (Tiangen). qRT-PCR was performed on a 7500 Real-Time PCR System (Applied Biosystems) using three biological replicates and three technical replicates per sample. Gene-specific primers (Table S10) were designed using Primer 5.0 (https://www.premierbiosoft.com/primerdesign/). Amplification efficiency was confirmed to be within 90%–110% using standard curves. Relative gene expression levels were calculated using the 2⁻^ΔΔCt^ method [54], with *Ubi* (*Zm00001d010159*) as the internal reference gene.

### Enzyme-linked immunosorbent assay (ELISA) for starch synthesis-related enzymes

The concentrations of four starch synthesis-related enzymes—AGPase, GBSS, SSS, and SBE—were quantified in endosperm tissues using commercial plant-specific ELISA kits (MEIKE Biotechnology, Jiangsu, China), based on principles similar to the double-antibody sandwich method. Assays were performed according to the manufacturer’s instructions and previously established procedures [53]. All four assays followed similar procedures, with kit-specific standards and reagents.

Briefly, fresh endosperm tissue was ground in liquid nitrogen and homogenised in phosphate-buffered saline (PBS; 0.01 mol/L, pH 7.2–7.4) at a ratio of 1 g tissue per 9 mL PBS. The homogenate was centrifuged at 8,000 × g for 30 min at 4 °C, and supernatants were collected and stored at 4 °C. Standards were serially diluted to generate a five-point standard curve. ELISA plates and reagents were equilibrated to room temperature (22–25 °C) for 20 min. Blank wells received 50 µL dilution buffer, standard wells received 50 µL of each standard solution, and sample wells received 50 µL extracted supernatant diluted 1:4. Plates were incubated at 37 °C for 30 min, liquids were discarded, and wells were washed five times with 150 µL wash buffer. Enzyme conjugate solution (50 µL) was added to each well, followed by incubation at 37 °C for 30 min, liquid removal, and repeated washing. Subsequently, chromogenic substrate (50 µL) was added and incubated at 37 °C for 15 min in the dark. The reaction was terminated by adding 50 µL stop solution, and absorbance was measured at 450 nm, with blank wells used as the zero reference. A regression equation was established based on the standard curve to calculate enzyme concentration in the test samples. All measurements were performed in duplicate.

### Phenotypic measurements

Plant height (PH) and ear height (EH) were measured at 15 DAP corresponding to physiological maturity (R6 stage) following Shi et al. [55]. PH was defined as the vertical distance from the soil surface to the tassel tip, and EH as the distance from the soil surface to the node bearing the primary ear. Measurements were obtained using a telescopic measuring rod (± 1 cm precision) for 10 randomly selected plants per row. For kernel morphological traits, 10 filled kernels were randomly selected from self-pollinated mature ears. Kernel length, width, and thickness were measured using a vernier calliper, with kernels arranged in tight alignment. Measurements were performed with six replicates.

## Supplementary Information

Supplementary Material 1. Table S1 Kernel starch content of 341 inbred lines assessed in 2018 and 2019 at Changping (Beijing) and Gongzhuling (Jilin). Table S2 GWAS-associated significant single nucleotide polymorphisms (SNPs) and genes. Table S3 Significant SNPs overlapping with QTL associated with starch content reported in previous studies. Table S4 Gene expression levels in maize endosperm at 20 days after pollination (DAP) for BSSS subpopulation lines. Table S5 Gene expression levels in maize endosperm at 20 days after pollination (DAP) for PA subpopulation lines. Table S6 Comparison of gene expression levels related to starch biosynthesis between BSSS and PA subpopulation lines. Table S7 The 29 transcription factors identified in GWAS associated with kernel starch content. Table S8 Correlation analysis among 29 transcription factors and starch biosynthesis genes. Table S9. Haplotypes of target transcription factors in different maize lines. Table S10. Gene-specific primers used in this study.

Supplementary Material 2. Fig. S1 Frequency distribution of kernel starch content within the maize-inbred population. (A) 2018CP, (B) 2018GZL, (C) 2019CP, (D) 2019GZL. Fig. S2 KEGG analysis of GWAS-associated genes. Pathways marked with blue boxes represent the top three. Fig. S3 Spatiotemporal expression patterns of ZmMYB71, ZmMYB4, and ZmGNAT16 in maize seed tissues. Fig. S4 Correlation analysis of transcription factors (TFs) and starch biosynthesis genes. TF–gene associations were assessed using Pearson’s correlation coefficient (PCC), followed by Mantel test validation. Fig. S5 Kernel starch content of maize inbred lines harboring pyramided favourable haplotypes of ZmMYB71, ZmMYB4, and ZmGNAT16. Different letters above the bars indicate significant differences in starch content associated with the number of favorable haplotypes, as determined by one-way ANOVA (*P* < 0.05).

## Data Availability

The complete datasets produced and analyzed throughout this investigation are fully accessible within the principal manuscript and its supplementary information files. The sequence data generated in this study have been deposited in the NCBI SRA database under accession number PRJNA1175378 and are available at the following URL: https://www.ncbi.nlm.nih.gov/search/all/?term=PRJNA1175378.

## Abbreviations

SC: Starch content
BSSS: Iowa stiff stalk synthetic (maize subpopulation)
PA: Patner A (maize subpopulation)
DAP: Days after pollination
SNP: Single nucleotide polymorphism
GWAS: Genome-wide association study
QTL: Quantitative trait locus
TF: Transcription factor
TPM: Transcripts per million
DEG: Differentially expressed gene
ELISA: Enzyme-linked immunosorbent assay
AGPase: ADP-glucose pyrophosphorylase
SSS: Soluble starch synthase
GBSS: Granule-bound starch synthase
SBE: Starch branching enzyme
