# Supplementary material for: Identification of transcription factors regulating starch biosynthesis in maize through integrated GWAS and transcriptomic analysis
Source: BMC Plant Biol. 2026 Mar 13;26:723. doi: 10.1186/s12870-026-08557-z (PMC13097711; doi:10.1186/s12870-026-08557-z)
Supplement: Supplementary file 2 — Supplementary Material 2. Fig. S1 Frequency distribution of kernel starch content within the maize-inbred population. (A) 2018CP, (B) 2018GZL, (C) 2019CP, (D) 2019GZL. Fig. S2 KEGG analysis of GWAS-associated genes. Pathways marked with blue boxes represent the top three. Fig. S3 Spatiotemporal expression patterns of ZmMYB71, ZmMYB4, and ZmGNAT16 in maize seed tissues. Fig. S4 Correlation analysis of transcription factors (TFs) and starch biosynthesis genes. TF–gene associations were assessed using Pearson’s correlation coefficient (PCC), followed by Mantel test validation. Fig. S5 Kernel starch content of maize inbred lines harboring pyramided favourable haplotypes of ZmMYB71, ZmMYB4, and ZmGNAT16. Different letters above the bars indicate significant differences in starch content associated with the number of favorable haplotypes, as determined by one-way ANOVA (P < 0.05). [file 12870_2026_8557_MOESM2_ESM.docx]

**Supplementary Figures**

**
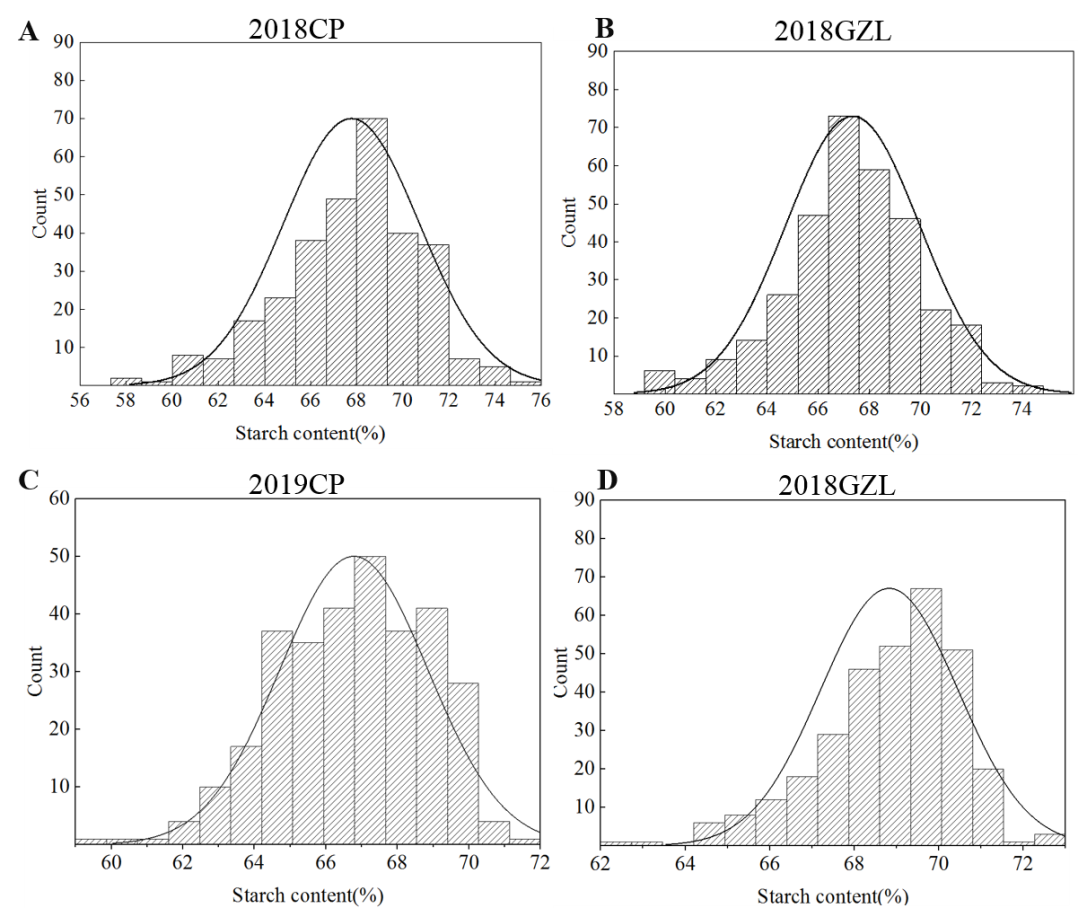
**

**Fig. S1 Frequency distribution of kernel starch content within the maize-inbred population.** (A) 2018CP, (B) 2018GZL, (C) 2019CP, (D) 2019GZL.


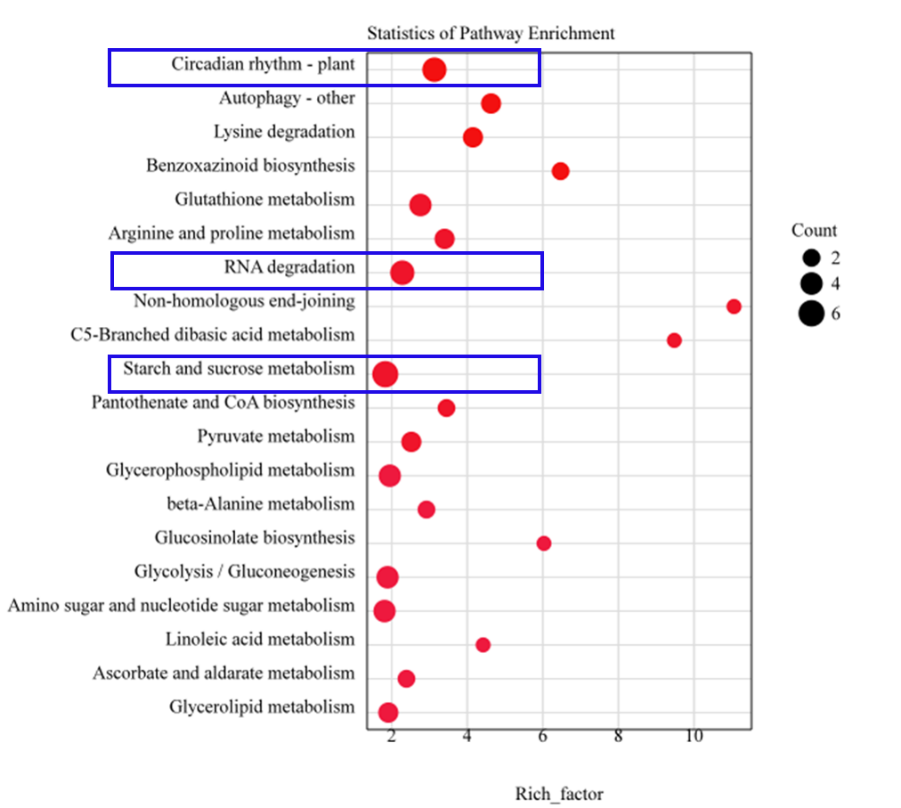


**Fig. S2 KEGG analysis of GWAS-associated genes. Pathways marked with blue boxes represent the top three.**


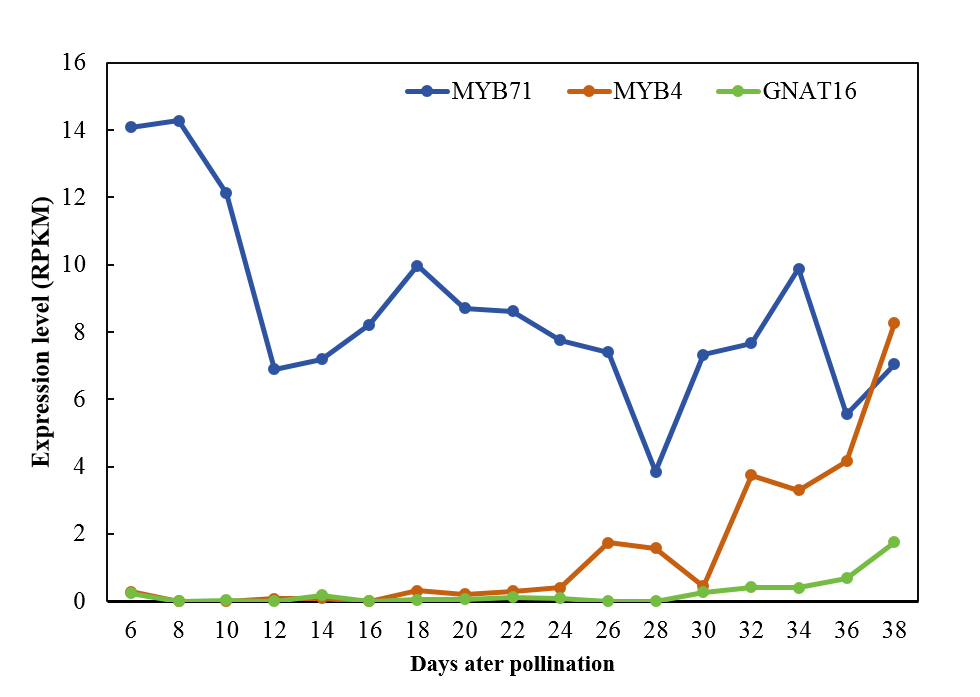


**Fig. S3 Spatiotemporal expression patterns of *ZmMYB71*, *ZmMYB4*, and *ZmGNAT16* in maize seed tissues.**


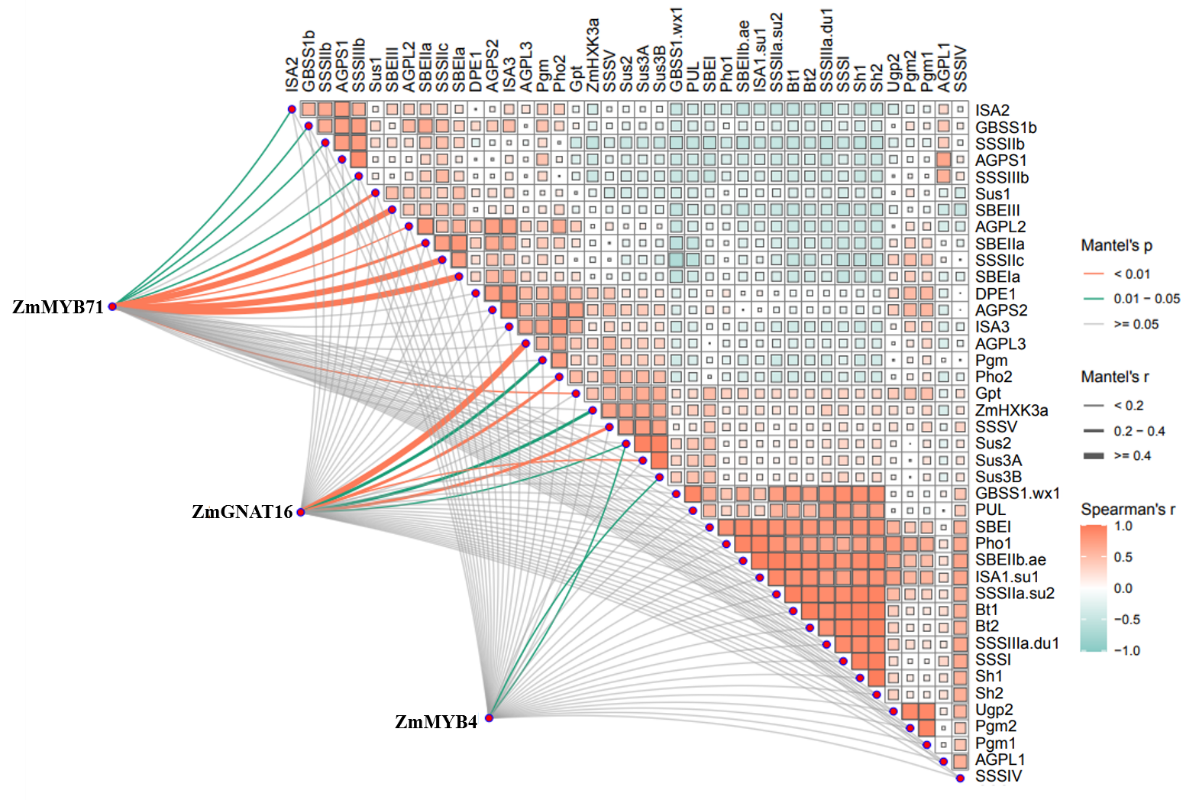


**Fig. S4 Correlation analysis of transcription factors (TFs) and starch biosynthesis genes.** TF–gene associations were assessed using Pearson’s correlation coefficient (PCC), followed by Mantel test validation.


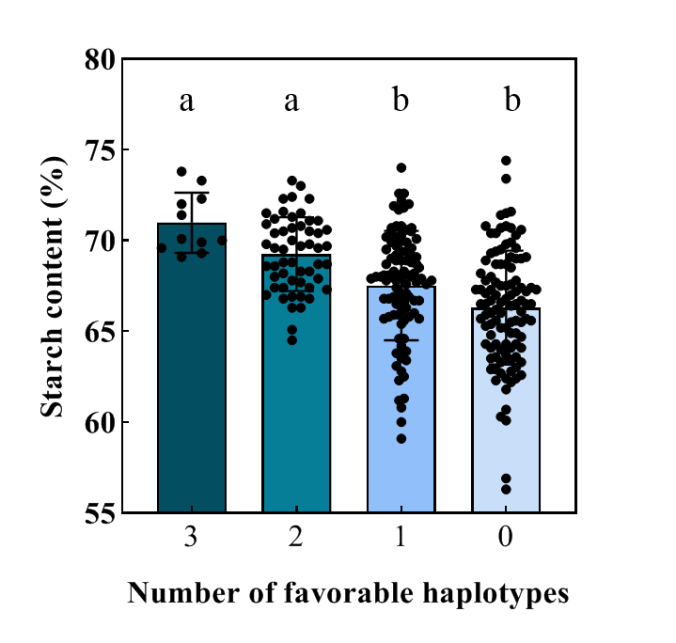


**Fig. S5 Kernel starch content of maize inbred lines harboring pyramided favourable haplotypes of *ZmMYB71*, *ZmMYB4*, and *ZmGNAT16*.** Different letters above the bars indicate significant differences in starch content associated with the number of favorable haplotypes, as determined by one-way ANOVA (*P* < 0.05).
